# Nonalcoholic Fatty Liver Disease (NAFLD): Pathogenesis and Noninvasive Diagnosis

**DOI:** 10.3390/biomedicines10010015

**Published:** 2021-12-22

**Authors:** Vicneswarry Dorairaj, Siti Aishah Sulaiman, Nadiah Abu, Nor Azian Abdul Murad

**Affiliations:** UKM Medical Molecular Biology Institute (UMBI), Universiti Kebangsaan Malaysia (UKM), Kuala Lumpur 56000, Malaysia; p105311@siswa.ukm.edu.my (V.D.); nadiah.abu@ppukm.ukm.edu.my (N.A.); nor_azian@ppukm.ukm.edu.my (N.A.A.M.)

**Keywords:** NAFLD, MAFLD, diagnosis, biomarkers, noncoding RNAs, extracellular vesicles

## Abstract

The global prevalence of nonalcoholic fatty liver disease (NAFLD) or metabolic associated fatty liver disease (MAFLD), as it is now known, has gradually increased. NAFLD is a disease with a spectrum of stages ranging from simple fatty liver (steatosis) to a severe form of steatosis, nonalcoholic steatohepatitis (NASH), which could progress to irreversible liver injury (fibrosis) and organ failure, and in some cases hepatocellular carcinoma (HCC). Although a liver biopsy remains the gold standard for accurate detection of this condition, it is unsuitable for clinical screening due to a higher risk of death. There is thus an increased need to find alternative techniques or tools for accurate diagnosis. Early detection for NASH matters for patients because NASH is the marker for severe disease progression. This review summarizes the current noninvasive tools for NAFLD diagnosis and their performance. We also discussed potential and newer alternative tools for diagnosing NAFLD.

## 1. Introduction

Nonalcoholic fatty liver disease (NAFLD), now known as metabolic associated fatty liver disease (MAFLD), is a common liver disease that affects 25% of the population worldwide [1,2]. Even though NAFLD is more prevalent among Hispanics, previous studies reported that NAFLD is increasingly becoming an issue in other populations [1,2]. Importantly, NAFLD has become more prevalent in children (~10%), particularly in children with obesity (34%) [3]. NAFLD is a disease with a broad spectrum of liver conditions without other known causes. The patients could progress from simple steatosis characterized by excessive hepatic triglyceride accumulation to a more severe form of fatty liver (nonalcoholic steatohepatitis, NASH) with and without fibrosis [4]. Although the majority of NAFLD patients will not progress, those with NASH and fibrosis are at risk of developing severe liver complications and mortality [5,6]. Thus, significant efforts are being made to understand the critical steps of NASH development from simple steatosis to fibrosis for developing early and accurate diagnostic tools for patient risk stratifications.

Currently, the gold standard assessment for NASH and fibrosis is a histological assessment of the liver (liver biopsy) [7]. However, liver biopsy is unsuitable for population screening due to its limitations, including the invasiveness that could lead to complications, such as bleeding, pain, and in some instances death [8]. As a result, there is an urgent need to address and develop alternative noninvasive diagnostic tools. Therefore, this review summarizes the current noninvasive methods for detecting NAFLD and discusses newer promising tools, including genetic approaches, noncoding RNAs, and extracellular vesicles (EVs).

## 2. NAFLD Pathogenesis

Understanding the pathogenesis of NAFLD is crucial for identifying the important molecular biomarkers crucial for accurate diagnosis. NAFLD is a multifactorial disease associated with unhealthy lifestyles and diets, metabolic dysregulation, genetics, oxidative stress, and altered gut–liver axis, all of which might influence disease development and progression. Detailed pathogenesis and molecular mechanisms have been described before [9].

Currently, the new proposed name for NAFLD is MAFLD. In the early 1980s, the term NAFLD was applied to patients with liver histological characteristics similar to those found in cases of alcohol-associated liver disease (ADL) yet the patients did not have heavy alcohol consumption [10]. Later, various publications showed that fatty liver is associated with type 2 diabetes mellitus (T2DM), obesity, and insulin resistance [10,11]. Thus, MAFLD is proposed as the new name for this liver condition, in which MAFLD accurately describes the liver manifestations of multi-metabolic disorders [10,12]. According to the current consensus, the diagnosis of MAFLD requires patients to have hepatic steatosis with any of the following metabolic disorders, such as obesity, T2D, and metabolic syndromes [10,12]. With the inclusion of metabolic disorders, different subtypes within the MAFLD patients are almost inevitable. Each subtype could have a different prognosis based on its pathophysiological progression. [10,12]. Since the MAFLD term was introduced recently, the diagnosis of MAFLD in clinical settings would require further investigation.

Dysregulation of the metabolic features drives early NAFLD/MAFLD disease development and steatosis. Overnutrition, insulin resistance, and obesity contribute multiple insults that modulate excess hepatic lipid accumulation [13]. Among the lipids or triglycerides (TG) in the liver, about 59% are from the circulating free fatty acids (FFAs) from adipocytes, followed by de novo liver lipogenesis (DNL) and dietary fats [14]. Lipolysis is a process of TG breakdown into FFAs by lipase enzymes to meet energy requirements. The activation of β-adrenergic leads to cyclic adenosine phosphate (cAMP) production [15]. These cAMPs bind to protein kinase A (PKA) and stimulate the phosphorylation of lipase enzymes [16]. Adipose triglyceride lipase (ATGL), currently known as patatin-like phospholipase domain containing 2 (PNPLA2), drives the first step in lipolysis. This PNPLA2 lipase hydrolyzes the ester bond of TG into diacylglycerol (DAG), and hormone-sensitive lipase mediates the hydrolysis of DAG to monoacylglycerol (MAG). Following this, the monoglyceride lipase catalyzes the hydrolysis of MAG to glycerol and FFAs [15]. Usually, circulating FFAs are higher during fasting and decrease upon feeding due to insulin signaling, suppressing lipolysis. However, in subjects with insulin resistance, higher lipolysis causes a more significant rise in circulating FFAs [9]. Mechanistically, upon insulin binding, insulin receptor substrate (IRS) is activated and phosphorylates phosphoinositide 3-kinase (PI3K) and protein kinase B (PKB), also known as AKT (PI3K/AKT pathway), to initiate insulin-mediated effects [15]. One of the enzymes activated in this pathway is phosphodiesterase 3B (PDE3B), and this enzyme catalyzes the hydrolysis of cAMP to inhibit lipolysis. In contrast, tumor necrosis factor-α (TNF) promotes lipolysis by p44/42/Jun kinases and thus inhibits insulin signaling [17]. Therefore, uncontrolled lipolysis due to insulin resistance in adipocytes highlights the role of adipocytes in liver steatosis. This finding is also supported by the increased rate of circulating FFAs associated with higher fat mass [18], thus further confirming that MAFLD is more accurate to describe the NAFLD condition.

Besides lipolysis, liver DNL also contributes to steatosis. DNL is a biochemical process that synthesizes FFAs from the acetyl-CoA subunits from glycolysis. The process starts with converting the acetyl-CoA to malonyl-CoA via acetyl-coenzyme A carboxylase (ACC) and finally to saturated fat, palmitate [19]. Two transcription factors regulate DNL. One is sterol regulatory element-binding protein 1c (SREBP1c), currently known as sterol regulatory element-binding transcription factor 1 (SREBF1) [19]. SREBF1 activation leads to the transcription of lipogenic genes, such as the ACC, stearoyl-CoA desaturase 1 (*SCD1*), fatty acid synthase (*FASN*), and the elongation of long-chain fatty acids family member 6 (*ELOVL6*) [20]. The other factor is the carbohydrate regulatory element-binding protein (ChREBP) [19]. In contrast to SREBF1, higher glucose uptake into the liver and glycolysis activate the ChREBP. Although the exact ChREBP mechanism is partly understood, the suggested mechanism is that hyperglycemia stimulates the transcriptional activity of ChREBP. Together with SREBF1, these transcription factors activate the downstream lipogenic genes and thus could explain the association of NAFLD with T2D or hyperglycemia.

Most NAFLD patients will not progress to NASH; however, those with NASH are at risk of developing severe liver diseases [5,6]. Even though the exact mechanism of NASH development is partly understood, lipotoxic and damaged hepatocytes could drive NASH progression [21,22]. In order to minimize lipid accumulation, the liver adapts to increase the disposal of FFAs via mitochondrial β-oxidation. However, this adaptation is lost in NASH individuals due to oxidative stress [23]. Increased reactive oxygen species (ROS) levels lead to reduced expression of peroxisome proliferator-activated receptor α (*PPARA*), which is a crucial transcriptional factor in FFA oxidation, thus causing the dysregulation of lipid oxidation [24,25]. Another feature is the liver inflammation that distinguishes NASH from steatosis. The adipose-derived cytokine TNF interferes with insulin signaling and contributes to hepatic inflammation [26]. The dysregulated metabolic molecules from steatosis, such as FFAs, cholesterol, oxidized low-density lipoproteins (OxLDLs), glucose, and advanced glycation end products (AGEs), could also initiate the pro-inflammatory mediators [27]. Notably, an animal model of NAFLD showed that hepatic resident macrophages (Kupffer cells) engulfed cholesterol crystals and became activated [28]. Activated Kupffer cells secrete TNF to amplify the effects of insulin resistance and activate the nuclear factor-κB (NFKB) and C-C motif chemokine ligand 2 (CCL2) [22]. Both NFKB and CCL2 are essential for activating the pro-inflammatory macrophages and monocytes to initiate liver inflammation [22]. Maintaining the inflammatory liver environment further activates the hepatic stellate cells (HSCs), a critical step for fibrosis development.

Hepatic fibrogenesis is driven by HSC activation and proliferation. Usually, HSCs are quiescent non-proliferative cells, and their activations lead to extracellular matrix (ECM) protein synthesis and production [29]. Lipotoxic hepatocytes and Kupffer cells could trigger HSC activation via the release of the pro-fibrotic cytokines (TNF, platelet-derived growth factor (PDGF), and transforming growth factor-β (TGFB)). The increase of α-smooth muscle actin (ACTA2) and desmin (DES) productions change the HSC phenotypes into proliferative and contractile shapes [29]. These activated HSCs also promote the secretion of pro-inflammatory cytokines, including CCL2 and interleukins (IL-6 and IL-8), to maintain the inflammatory environment and promote the fibrogenic environment further [30]. Thus, as collagen deposition becomes more evident in liver tissue, the patients have progressed to cirrhosis. Therefore, understanding NAFLD pathogenesis will allow for an accurate diagnosis for early intervention.

## 3. Current Noninvasive Diagnostic Methods

Current noninvasive methods for detecting NAFLD focus on the two elements: (1) quantification of serum or plasma biomarkers and (2) measurement of liver stiffness via imaging techniques, such as ultrasound- or magnetic resonance-based tools.

### 3.1. Serum Biomarkers

Most serum or blood biomarkers are incorporated into predictive models to diagnose NAFLD (Table 1). One such model is the diagnosis of steatosis index that includes the fatty liver index [31], hepatic steatosis index [32], SteatoTest [33], lipid accumulation product (LAP) [34], index of NASH (ION) [35], NAFLD liver fat score (LFS) [36], triglyceride-glucose index (TyG) [37], serum keratin 18 fragment (CK-18) [38], and visceral adiposity index (VAI) [39]. These index models’ diagnostic performance is acceptable; however, the performance is suboptimal when it comes to distinguishing steatosis grades [31,32,33,34,35,36,38]. Moreover, these indexes cannot differentiate among NAFLD individuals with and without NASH [40].

**Figure 1:**
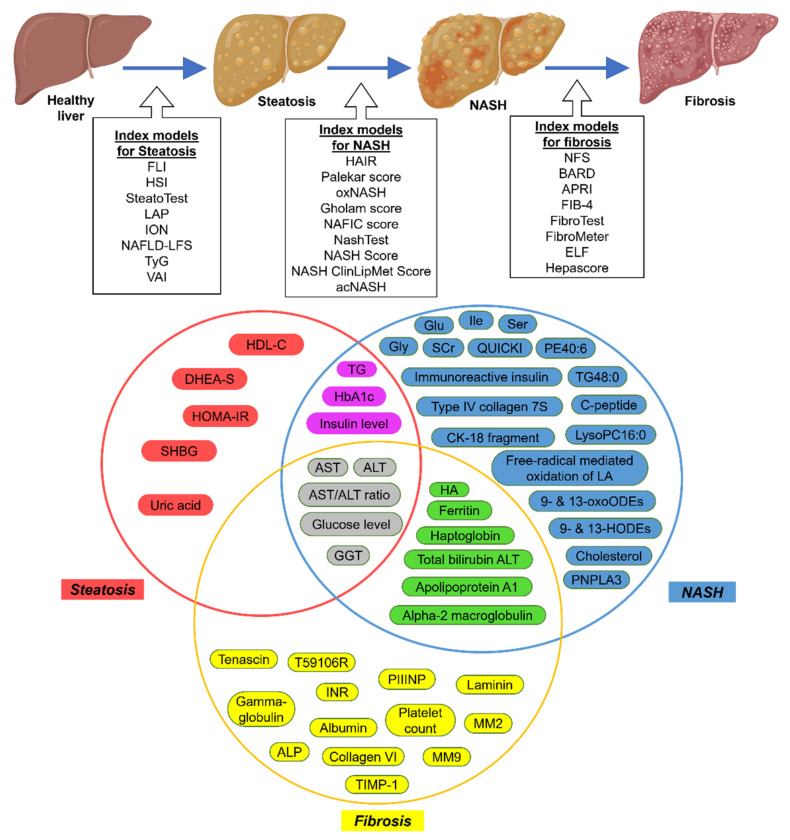
Graphical representation of the NAFLD indexes and the overlapping molecules. Abbreviation: ALT: alanine transaminase; ALP: alkaline phosphatase; APRI: aspartate transaminase-to-platelet ratio index; AST: aspartate transaminase; BMI: body mass index; DHEA-S: dehydroepiandrosterone sulphate; DBP: diastolic blood pressure; FP: fasting plasma; FLI: fatty liver index; GGT: gamma-glutamyltransferase; HbA1c: hemoglobin A1c; Glu: glutamate; Gly: glycine; glycosylated hemoglobin A1c; HA: hyaluronic acid; HDL-C: high-density lipoprotein cholesterol; HIS: hepatic steatosis index; HODE: hydroxy-octadecadenoic acids; HOMA-IR: homeostasis model assessment of insulin resistance; Ile: isoleucine; IRI: immunoreactive insulin; LA: linoleic acid; LAP: lipid accumulation product; LysoPC16:0: lysophosphatidylcholine; MetS: metabolic syndrome; MM: matrix metalloproteinase; NFS: NAFLD fibrosis score; NAFLD-LFS: NAFLD liver fat score; NASH: nonalcoholic steatohepatitis; oxoODE: oxo-octadecadenoic acids; PCOS: polycystic ovary syndrome; PE40:6: phosphoethanolamine 40:6; PIIINP: N-terminal propeptide of type III collagen; QUICKI: quantitative insulin sensitivity check index; SBP: systolic blood pressures; SCr: serum creatinine; Ser: serine; SHBG: sex hormone binding globulin; TG: triglycerides; TIMP-1: tissue inhibitor of matrix metalloproteinase 1; TyG: triglyceride-glucose index; VAI: visceral adiposity index; WC: waist circumstances.

Other serum biomarkers are for the diagnosis of NASH (Table 1). One such is circulating CK-18 levels, which could differentiate between patients with NASH and those with steatosis [58], though its performance is moderate [59]. Similar to steatosis, most serum or clinical biomarkers are incorporated into the predictive models to identify NASH, such as HAIR [41], Palekar score [42], oxNASH [43], Gholam score [44], NAFIC score [45], NashTest [46], NASH Score [47], NASH ClinLipMet Score [48], and acNASH [49]. Moreover, some of these serum markers overlap with those in the steatosis indexes (Figure 1). Unfortunately, none of these NASH indexes could differentiate NASH from steatosis with high sensitivity and specificity.

Other models or indexes are intended for diagnosing and grading fibrosis (Table 1 and Figure 1). Examples are the NAFLD fibrosis score (NFS) [50] and the BARD score [51] that are more specific to NAFLD whereas the other indexes were developed originally to diagnose hepatitis patients, e.g., the aspartate transaminase-to-platelet ratio index (APRI) [52], FIB-4 [53], FibroTest [54], and proprietary indexes, such as the FibroMeter [55], enhanced liver fibrosis (ELF) [56], and Hepascore [57]. Of these indexes, NFS and FIB-4 are the most accurate, with high sensitivity for identifying individuals without advanced fibrosis, thus eliminating those patients that do not need further assessment [60]. Moreover, FIB-4 is more favorable because its formula only uses simple parameters readily available from the standard clinical reports [60]. Unfortunately, both indexes require extra investigations if the individuals are positive for advanced fibrosis, and in some cases (~30%) the diagnosis is unclear even with these indexes [61]. A newly identified plasma marker, Pro-C3 (N-terminal type III collagen propeptide), is reported to be more reliable than the existing indexes (FIB-4, APRI, and NFS) for identifying individuals with NAFLD and advanced fibrosis [62], though this finding requires further validation with a larger cohort. Serum or plasma markers are easy means of diagnosing NAFLD in clinical settings and are often used together with ultrasound techniques to confirm the diagnosis. For example, the FLI index combined with ultrasound as a reference is commonly used to diagnose steatosis, though this practice has moderate sensitivity. Another option is the HIS index combined with ultrasound reference, though the accuracy is still sub-optimal. The NAFLD-LFS is superior to FLI and HIS because it uses proton magnetic resonance spectroscopy (H-MRS) as a reference. Since H-MRS is not standard clinic equipment, FLI and HIS indexes with ultrasound remain the recommended option for diagnosing steatosis [63]. As for fibrosis, most indexes are generally accurate in diagnosing advanced fibrosis. NFS and FIB-4 are the most recommended and commonly used to screen individuals without significant fibrosis due to their high sensitivity. Thus, these indexes are used as triage in primary care [63]. Unfortunately, these indexes cannot differentiate the individuals with NASH. Identification of NASH-specific markers is vital as the presence of NASH determines the worst outcome in the patients.

### 3.2. Imaging-Based Techniques

Besides the serum or blood markers, most NAFLD clinical assessments require imaging-based techniques, such as elastography, to confirm the diagnosis. There are two elastography-based tools: ultrasound- and magnetic resonance-based [60]. The ultrasound (US) tool is the most commonly used and recommended for diagnosing NAFLD and steatosis [60]. The US abdominal image shows echogenicity—the ability to reflect US waves—allowing for visual contrasts between the liver and kidneys and observation of the intrahepatic vessels, liver parenchyma, and diaphragm [60]. Most conventional abdominal US detects the echogenicity of the liver and grades the steatosis into three stages: (1) grade 0 steatosis with less than 5% of fat-laden hepatocytes, (2) grade 1 steatosis with 6–33% of fat-laden hepatocytes, (3) grade 2 steatosis with 34–66% fat-laden hepatocytes, and (4) Grade 3 steatosis with more than 66% of fat-laden hepatocytes [64]. Although this method is commonly used due to its low cost and quick diagnosis, in a meta-analysis of 34 studies, the pooled sensitivity and specificity of conventional US for diagnosing steatosis stages (moderate and severe) were 85% and 93%, respectively [65]. However, in individuals with obesity, these accuracies are reduced [66]. Thus, the sensitivity of conventional US to detect steatosis is compromised when the degree of steatosis is less than 20% and has limited use in overweight and obese individuals [64].

A quantitative US tool uses the speed of shear waves in the liver tissue and converts this speed into a liver stiffness measurement (LSM) in kilopascals (kPa) [60,67]. The most commonly used quantitative US tool is transient elastography (TE); other tools are acoustic radiation force impulse imaging (ARFI) and strain elastography (SE) [67]. TE, more commonly known as Fibroscan (Echosens, Paris, France), is a vibration-controlled TE device that uses the controlled attenuation parameter (CAP). CAP measures the attenuation of US waves crossing the liver tissue to determine the presence of steatosis and its grades. CAP is often evaluated together with LSM and is available on both M and XL probes of the Fibroscan system [67,68,69]. In a meta-analysis of 19 studies and 2735 NAFLD patients, the CAP on the M-probe optimal cut-off values for different steatosis grades were 248 (237–261) dB/m for mild steatosis for above grade 0 steatosis, 268 (257–284) dB/m for significant steatosis (above grade 1), and 280 (268–294) dB/m for severe steatosis (above grade 2) [70]. However, the CAP only moderately differentiated steatosis grades (≥11%, ≥33%, and ≥66%) with area under the receiver operating curve (AUROC) scores of 0.82, 0.86, and 0.88, respectively [70]. The plausible reasons for these moderate accuracies are that several covariates could influence the CAP values, including NAFLD stage, diabetes, and body mass index (BMI) [70]. Notably, the CAP on the M-probe often overestimated liver fibrosis in individuals with steatosis [71]. Some studies showed that this limitation could be eliminated by using the CAP on the XL probe [72], though the CAP values on both probes give similar readings [73,74]. Nevertheless, only two studies reported the usage of the XL probe; therefore, more studies are needed to confirm this. Other US manufacturers also developed their proprietary technology to quantify the attenuation of the US wave. One example is the Canon Medical Systems (Tochigi, Japan) that uses attenuation imaging (ATI) in the Aplio i800 US systems [75]. In this ATI, the attenuation coefficient is calculated in decibels per centimeter per megahertz (dB/cm/MHz) and is displayed in a real-time color-coded map. Previous studies investigated the diagnostic potential of ATI compared to CAP and found that ATI offers slightly better accuracy in the grading of steatosis [76,77,78].

Another imaging technique to diagnose steatosis is magnetic resonance-based elastography (MRE) [60]. A meta-analysis of eight studies reported that MRE pooled sensitivity and specificity were 89% and 84%, respectively, and the AUROC to diagnose steatosis was 0.92 [79]. Like ultrasound, adding the proton-density fat fraction (PDFF) in magnetic resonance imaging (MRI) makes steatosis grading possible. The MRI-PDFF performs better than CAP in diagnosing all grades of steatosis [80]. Three studies, including American [81], Japanese [82], and Dutch [83] populations, showed that MIR-PDFF has better AUROC than the CAP-ultrasound. At present, neither MRE nor ultrasound tools could reliably differentiate NASH from simple steatosis. The MRI-based tool shows some potential for overcoming this problem, as the new LiverMultiScan (Perspectum Diagnostics) could distinguish NASH individuals (AUROC: 0.80) from simple steatosis cases [84]. However, this finding requires further validation.

In terms of fibrosis and cirrhosis, Fibroscan/TE has a range of good-to-excellent accuracies for diagnosing advanced fibrosis and cirrhosis. A meta-analysis of nine studies using the M-probe to diagnose advanced fibrosis and cirrhosis showed that the pooled sensitivity and specificity were 85% and 92%, respectively, for both fibrosis and cirrhosis [85]. Another meta-analysis of 19 studies (four studies using the XL-probe) reported that the AUROC between the M- and XL-probes for diagnosing advanced fibrosis and cirrhosis do not differ [86]. Nonetheless, TE is still the most recommended tool to diagnose NAFLD and fibrosis. As the TE has a 94–100% negative predictive value, it can rule out the individuals with no fibrosis with high accuracy [87]. Besides TE, MRI-based tools can also diagnose or detect fibrosis. Two meta-analysis studies showed that the 2D-MRE has a high diagnostic performance in detecting advanced fibrosis (AUROC: 0.93 and 0.96, respectively) [86,88]. Moreover, the diagnostic performance of 3D-MRE is even better than 2D-MRE (AUROC: 0.96 vs. 0.92, respectively). However, 3D-MRE takes a long time to process results [89]. Another MRI-based tool is the application of acoustic radiation force impulse imaging (ARFI) for diagnosing fibrosis and cirrhosis. A systematic review and meta-analysis of 29 studies revealed that the ARFI has high diagnostic accuracy for diagnosing advanced fibrosis with pooled sensitivity and specificity of 92% and 85%, respectively, and AUROC of 0.94 [90]. The above tools are excellent for diagnosing severe or advanced fibrosis, yet suboptimal for detecting early fibrosis. It is also important to note that most of these noninvasive tools for diagnosing NAFLD, NASH, and fibrosis are not optimized for the presence of type 2 Diabetes (T2D) [60]. Since individuals with T2D are at risk for NAFLD and advanced fibrosis [91], optimization and validation are needed to assess the actual accuracy of these imaging techniques in T2D individuals. At present, most imaging techniques (US and MRI-based) are excellent for detecting the presence of steatosis and fibrosis and their grading. However, the accuracies are compromised when the steatosis grade is too low (grades 0–1) and the patients have metabolic syndromes (T2D and obesity). Recent reports suggested that MRI-PDFF is superior among the imaging techniques (AUC: 0.946) in detecting hepatic steatosis in clinical settings [92]. Unfortunately, none of these imaging techniques could differentiate between NASH and the early fibrosis stage; thus, liver biopsy remains the gold-standard method.

## 4. Alternative Diagnostic Tools

### 4.1. Genetics of NAFLD

Another potential biomarker or alternative diagnostic tool is provided by the genetics of NAFLD. Previous genome-wide association studies (GWAS) reported several genetic variants associated with NAFLD risk [93,94]. The single nucleotide polymorphism (SNP) of the patatin-like phospholipase domain-containing 3 (*PNPLA3*) gene, rs738409 (C > G), results in a missense variation (I148M) which inhibits this enzyme’s activity and subsequently causes higher hepatic fat accumulation (75% higher) [95,96]. The *PNPLA3* gene encodes the lipid droplet-associated repressor that binds competitively to the co-activator of ATGL, thus causing higher lipid accumulation [94,97]. Individuals with variant G nucleotide have a 3.2-fold greater risk of developing hepatic fibrosis, and NASH is more prevalent in GG individuals than CC (odds ratio: 3.49) [96]. Moreover, a meta-analysis of 13,817 individuals showed that the I148M variant pooled odds ratio for NASH was 2.54. The odd ratios according to the genotypes were 1.75 for heterozygotes and 4.44 for homozygotes [98]. The I148M variant has become the most significant genetic determinant of NAFLD in various populations currently [93,94]. Moreover, the penetrance of this variant in the European population is comparable to monogenic liver disease mutation effects, with the homozygous GG having a high odds ratio (12.19) of developing HCC in NAFLD patients [99,100].

The frequency of the *PNPLA3* I148M variant significantly correlated with ethnicity and population prevalence of NAFLD. The I148M variant is relatively common, with a frequency of 26% (combined population). This I148M frequency is much higher for Hispanics (49%) and lowest for Africans (12–17%) [95]. Consistent with these frequencies, the Hispanics have higher NAFLD prevalence (45%), whereas the lowest NAFLD prevalence is in the Africans (24%) [1], thus suggesting that the variant I148M may explain the variability in hepatic steatosis between the different ethnic groups. Important findings relating to the *PNPLA3* genetic risk are that this variant I148M effect was independent of insulin resistance and could be modulated by dietary conditions [95]. Individuals with I148M have higher liver fat levels, but no effect was observed in their glucose tolerance, liver enzymes, and C-reactive proteins [95]. However, Hispanic children with this variant have higher liver fats when they have carbohydrate-rich diets [101]. The ChREBP transcription factor regulates *PNPLA3* expression, and high levels of carbohydrates activate the ChREBP transcription factor to facilitate lipid metabolism and regulation [102]. Thus, the disruption of the enzyme activity by the variant I148M confers susceptibility in the individuals when consuming carbohydrate-rich diets, suggesting a genetic and nutritional relationship. Importantly, this SNP is incorporated in the predictive model to diagnose NASH [47,48], indicating its potential as a biomarker for NAFLD and NASH.

Besides the *PNPLA3* gene, previous GWAS studies also reported other genes associated with NAFLD. One such is the transmembrane 6 superfamily, member 2 (*TM6SF2*) gene and its SNP rs58542926 (G > A) that results in a variant E167K. This variant was linked with higher liver triglyceride levels and a greater risk of having advanced fibrosis [103,104,105]. In contrast, this variant also associates negatively with the level of liver triglyceride-rich lipoproteins, thus causing a low risk of cardiovascular disease (CVD) [105,106]. The *TM6SF2* gene encodes an endoplasmic reticulum (ER) transmembrane protein. A loss of this protein function causes lower secretion of very low-density lipoprotein (VLDL) and increases hepatic lipid accumulation [107], thus partly explaining the low risk of CVD. Other reported GWAS genes are the glucokinase regulatory protein (*GCKR*) gene and its genetic variant SNP rs780094 [108,109], membrane-bound O-acyltransferase domain-containing 7 (*MBOAT7*) and its SNP rs641738 [110,111], and hydroxysteroid 17β-dehydrogenase (*HSD17B13*) SNP rs72613567 [112,113,114]. The latter SNP is protective and reduces NAFLD risk [115]. The *HSD17B13* variant rs72613567 is a splice variant at the last exon, causing a truncated mRNA transcript and loss of function [115]. Although the role of the HSD17B13 enzyme is partly understood, this variant rs72613567 causes the reduction of lipid droplets and chronic liver injury with no effect on hepatic steatosis [116]. Notably, there is a relationship between the *HSD17B13* rs72613567 variant and the *PNPLA3* I148M variant. In individuals carrying the *PNPLA3* I148M variant, the *HSD17B13* rs72613567 variant lowered the effects of the I148M variant on livery injury and hepatic enzyme levels [115,117,118]. Since the *HSD17B13* gene is primarily expressed in the liver [119], this genetic alteration could potentially be a therapeutic target for NAFLD. From the GWAS studies, the genetics of NAFLD have significant potential as a diagnostic tool. Among these genetic variants, the screening of *PNPLA3* I148M could identify individuals at risk for developing NAFLD as early as 3.1 years [120]. Moreover, the genotype of *PNPLA3* I148M is included in two clinical indexes, the NASH score and the ClinLipMet score, with AUROCs of 0.778 and 0.866, respectively [48]. Cumulative genetic risk scores (GRS) comprising *PNPLA3*, *TM6SF,* and *HSD17B13* variants predicted a 12-fold higher risk of cirrhosis and up to a 29-fold higher risk of HCC in 445,452 individuals [121]. Although genetic screening could diagnose early, the implementation of genetic screening is still not currently recommended by the American Association for the Study of Liver Diseases for clinical settings [122], though this may change in the future [123].

### 4.2. Noncoding RNAs in NAFLD

Recently, studies have indicated that noncoding RNAs (ncRNAs) could regulate NAFLD progression (Table 2) [124,125,126]. The ncRNAs are RNAs that do not encode functional proteins and are generally grouped based on their sizes, (1) small ncRNAs (microRNAs) and (2) large ncRNAs, including the long noncoding RNAs and circular RNAs. Some of these ncRNAs are stably present in the circulating samples, such as blood and urine, and therefore have enormous potential to be biomarkers for NAFLD.

#### 4.2.1. MicroRNAs

Among ncRNAs, microRNAs (miRNAs) are the most well-known in the context of NAFLD (Table 2) [177]. MiRNAs are small, single-stranded ncRNAs (~22 nucleotides) that negatively regulate gene expression by complementary binding to messenger RNAs (mRNAs). The majority of these miRNAs are transcribed from their genes during the canonical pathway centered around the microprocessor complexes of Drosha and Dicer. The detailed biogenesis of miRNAs has been described recently [178]. One miRNA, miR-122, is highly expressed in the liver, and this miRNA is known to maintain a healthy liver and function [125,177]. Low miR-122 expression in the hepatocytes leads to steatohepatitis, lowers plasma cholesterol levels, reduces fatty acid (FA) synthesis, and increases FA oxidation. Consistently, miR-122 regulates the genes involved in lipid and cholesterol metabolism, such as *SREBF1*, diacylglycerol O-acyltransferase 2 (*DGAT2*), *FASN*, and prolyl 4-hydroxylase subunit alpha 1 (*P4HA1*) [127]. Moreover, this low miR-122 expression was more evident in the liver of NASH individuals (10-fold lower) when compared to steatotic liver cases [177]. In contrast, serum miR-122 expression was higher in NAFLD patients than healthy controls and was much higher in NASH individuals [179]. Since this miRNA is preferably localized near the membrane of lipid-rich hepatocytes [92], high serum miR-122 may come from damaged hepatocytes [180]. Thus, a change of miR-122 expression could reflect the status of the liver organ, and therefore could be used as a biomarker for NAFLD progression.

Besides miR-122, other miRNAs also regulate steatosis and lipid metabolism. One such is the miR-21 that is higher in liver tissue and circulating plasma of NAFLD individuals and animal models [132,133]. Inhibition of the miR-21 expression alleviated steatosis by upregulation of the key regulators of lipid metabolism, such as hepatocyte nuclear factor 4 alpha (*Hnf4a*), forkhead box transcription factors (*Foxa2* and *Foxo1*), *Ppara*, and the signal transducer and activator of transcription 3 (*Stat3*) [134,135]. Another miRNA is the miR-34a that is also higher in the liver tissue of NAFLD animal models [139,140]. Similar to miR-21, the inhibition of miR-34a alleviated steatosis by the upregulation of *Ppara* and sirtuin 1 (*Sirt1*) expression [139,140]. One of the most abundant miRNAs in the liver is miR-99a, which was reduced in the serum samples of NAFLD individuals [181]. This miRNA is involved in the negative regulation of inflammatory signals by targeting the TNF [141] and mammalian target of rapamycin (mTOR)/SREBF1 [142]. The miR-33 family members, miR-33a and miR-33b come from the introns of *SREBF1* and *-2* genes [138]. SREBF1 regulates the genes for fatty acid synthesis, and SREBF2 regulates cholesterol metabolism [182]. Therefore, inhibition of the miR-33a increases cholesterol transporter (*ABCA1*) expression, HDL production, and circulating levels [182], suggesting that miR-33 plays a role in dyslipidemia. This finding is partly correct, as the levels of plasma miR-33 members were found to be higher in familial hypercholesterolemia children compared to a healthy group. Their levels also correlated with cholesterol, LDL and LDL/HDL ratio, and APOB levels [183], confirming their roles in lipid metabolism.

Another liver-specific miRNA is miR-192, mainly implicated in fibrosis via the TGFB/SMAD pathway [184]. Following liver injury, the activation of the TGFB1 signal decreased the binding of hepatocyte nuclear factor family factors to the promoter region of miR-192 and subsequently reduced this miRNA expression [185]. Therefore, this miR-192 inhibition increases the expression of its targets such as epiregulin (*EREG*), activated leukocyte cell adhesion molecule (*ALCAM*), and moesin (*MSN*), and these molecules are involved in epithelial-to-mesenchymal transitions (EMT) [130]. A study of an animal model of liver injury supported this finding, as miR-192 also negatively regulates zinc finger E-box binding homeobox 2 (*Zeb2*) expression. This Zeb2 is a well-known EMT modulator [131], critical for fibrosis formation. Similar to miR-122, circulating miR-192 was found to be higher in NASH patients [186] and could be used as a biomarker for NASH.

These circulatory miRNAs are used in a diagnostic panel to identify and diagnose NAFLD. A panel of five serum miRNAs, miR-122, miR-34a, miR-375, miR-192, and miR-21, was specific enough to differentiate NAFLD individuals from drug-induced liver injury cases [187]. A systematic review and meta-analysis revealed that from 27 studies three miRNAs (miR-122, miR-34a, and miR-99a) could significantly diagnose NAFLD, with pooled AUROC results of 0.86, 0.85, and 0.87, respectively [188]. Importantly, miR-34a has the lowest heterogeneity and thus has the highest potential as a biomarker for NAFLD [188]. This panel of miRNAs (miR-122, miR-34a, and miR-99a) has a greater AUROC of 0.91 to differentiate NASH from simple steatosis. Moreover, this miRNA panel accuracy is the best when the individuals have a body mass index (BMI) of more than 30kg/m^2^ [188]. Another recent study of the Korean population showed that a panel of four miRNAs (miR-192-5p, miR-21-5p, miR-4449, and miR-151a-3p) also have moderate AUROC (0.875) to distinguish NASH individuals from simple steatosis individuals [189]. Although miRNAs could diagnose NASH, more studies are needed to validate these findings for clinical settings.

#### 4.2.2. Long Noncoding RNAs

Similar to miRNAs, long noncoding RNAs (lncRNAs) also do not produce protein products, and they could regulate multiple processes in NAFLD [190]. LncRNAs could regulate gene expression in a manner of cis- or trans-regulation via epigenetic regulation, chromatin remodeling, and transcriptional and post-transcriptional regulations. Mechanistically, the lncRNAs act as (1) scaffolds or platforms to provide a site for molecular interactions, (2) decoys to prevent protein interactions, and (3) sponges to compete for the binding of RNA molecules (miRNAs) and prevent their downstream actions [191]. Since previous reviews have comprehensively discussed the biogenesis, classification, functions, and roles of lncRNAs in NAFLD [190], only selected and most significant lncRNAs are discussed here.

A few lncRNAs regulate hepatic steatosis (Table 2). One such is the lncRNA H19 imprinted maternally expressed transcript (*H19*), which is one of the essential lncRNAs in hepatic steatosis. In a study of H19 knockout mice, *H19* lncRNA acts as a scaffold to facilitate the interaction between the polypyrimidine tract binding protein 1 (Ptbp1l) and Srebf1, thus activating downstream lipogenesis genes [146]. In another study of high-fat diet mice, *H19* lncRNA acts as a sponge to miR-130a to upregulate peroxisome proliferator-activated receptor gamma (*Pparg*) expression, concomitant with hepatic steatosis [192]. The miR-130a inhibits hepatic steatosis by suppressing the expression of NAFLD-related genes, including *Pparg*, *Srebf1*, *Scd1*, *Acc1*, and *Fasn* [192]. Therefore, the interaction between *H19* and miR-130a to regulate *Srebf1* could be the primary driver for hepatic steatosis. Another lncRNA is the steroid receptor RNA activator (*SRA*). This lncRNA negatively regulates the expression of *Atgl* and promotes hepatic steatosis via the suppression of fork-head box protein O1 (*Foxo1*) and *Pparg* which promotes the transactivation of the Atgl enzyme. Silencing *SRA* expression in the mice restored *Atgl* expression, prevented FA oxidation, and relieved hepatic steatosis [159]. Besides *SRA*, the metastasis-associated lung adenocarcinoma transcript 1 (*MALAT1*) lncRNA is an inflammatory lncRNA associated with diabetic conditions [193]. In animal and cell models of NAFLD, *MALAT1* expression was higher, and this upregulation caused an increase of *Srebf1* mRNA and protein expression and promoted hepatic steatosis [156]. Further investigation revealed that *MALAT1* interacts with the Srebf1 protein to stabilize it, thus leading to lipid lipogenesis gene activations [156]. In contrast to the above lncRNAs, a specific liver lncRNA, lncRNA suppressor of hepatic gluconeogenesis and lipogenesis (*lncSHGL*, also known as *B4GALT1-AS1*, the human homolog) is a protective lncRNA for hepatic steatosis [155]. This lncRNA suppressed fatty liver accumulation and hyperglycemia in high-fat diet mice by recruiting the heterogeneous nuclear ribonucleoprotein A1 (Hnrnpa1) to increase the rate of calmodulin (Cam) protein translation [155]. This CAM protein suppresses the mTOR/SREBF1 pathway and activates the PI3K/AKT pathway, alleviating hyperglycemia and hepatic steatosis [155,194]. This lncRNA *B4GALT1-AS1*/Hnrnpa1/Cam axis could be used as a therapeutic target for individuals with NAFLD and T2D.

Some lncRNAs regulate hepatic fibrosis. One such is liver-specific lncRNA, liver fibrosis-associated lncRNA 1 (*LFAR1*), and this lncRNA expression was higher in the animal model of fibrosis [152]. *LFAR1* acts as a scaffold to allow for the binding of the Smadfamily protein complex, Smad2/Smad3, to the receptor of Tgfb1, in turn activating the downstream fibrosis-related genes in TGFB signaling [152]. Another lncRNA is the HOX transcript antisense RNA (*HOTAIR*). This lncRNA expression is higher in the animal and cell model of fibrosis [195]. Mechanistically, *HOTAIR* acts as a sponge to miR-148b, a known negative regulator of the DNA methyltransferase 1 (*DNMT1*) [148]. In this study of both human and mouse hepatic stellate cell (HSC) lines [148], an increase of *DNMT1* expression leads to hypermethylation on the promoter region of another lncRNA, maternally expressed 3 (*MEG3*), and suppressed this lncRNA expression. Furthermore, *HOTAIR* also acts as a scaffold at the *MEG3* promoter region and recruits the polycomb repressive complex 2 (PRC2) to initiate H3K27 methylation, further suppressing *MEG3* gene transcription [148]. *MEG3* is anti-fibrotic lncRNA which increases tumor suppressor p53 (*TP53*) expression to initiate HSCs apoptosis [157], thus preventing the activation and proliferation of HSCs upon the TGFB1 signal. Additionally, MEG3 acts as a sponge to miR-21, a regulator of cholesterol metabolism [158]. The suppression of miR-21 action increased the expression of LDL receptor-related protein 6 (*LRP6*), thus alleviating lipid accumulation [158]. Another reported lncRNA is the Alu-mediated p21 transcriptional regulator (*APTR*), a recently recognized cell cycle and proliferation regulator [143]. High expression of *APTR* was observed in animal models and human patients with fibrosis [143]. Silencing of *APTR* expression prevented an accumulation of extracellular matrix protein (ECM) and HSCs activation [143].

The lncRNAs are also incorporated into panels for diagnosing NAFLD and NASH. Although the data are still limited, one study investigated the diagnostic potential of serum expression of *Lnc-SPARCL1-1:2* in NAFLD. In this study [196], *Lnc-SPARCL1-1:2* has moderate sensitivity and specificity to distinguish NASH individuals from healthy controls and NAFLD with steatosis. However, this lncRNA could significantly identify NASH individuals from NAFLD individuals who do not have steatosis, with AUROC of 0.974, a sensitivity of 90%, and a specificity of 100% [196]. Similarly, another study used the plasma expression of lncRNA *TCONS_00016452/LEXIS* to identify the NASH individuals. In this study [197], the expression of *LEXIS* was higher in NASH than simple steatosis individuals, but the diagnostic performance was moderate with an AUROC of 0.743. In another study of NAFLD individuals, the ratio of serum expression of *TGFB2* to its associated lncRNAs, *TGFB2-OT1*, was included in a panel together with FIB-4 markers, and this panel was able to identify fibrosis with high accuracy (AUROC: 0.891) [198]. In the same study [198], this ratio of *TGFB2/TGFB2-OT1* in a panel with Fibroscan was also able to identify fibrosis in individuals with similar AUROC. For NASH identification, the serum expression of lncRNA *RP11-128N14.5* has similar diagnostic potential with serum AST level; despite that, this lncRNA expression was higher in NASH individuals [198]. Furthermore, a combination of lncRNA *RP11-128N14.5* expression with the clinical markers did not improve the diagnostic performance. Therefore, the possibility of identifying novel NASH-specific lncRNAs as biomarkers for early diagnosis is still open, and it is still to be determined whether these lncRNAs could be used in primary care settings.

#### 4.2.3. Circular RNAs

Circular RNAs (circRNAs) are large ncRNAs that do not encode for functional proteins and are the products of the transcription process, though circRNAs are derived from the back-splicing events [199,200,201]. This back-splicing process produces covalently closed loop RNAs that contain either exons or introns or mixtures of both, with no 5′-cap or 3′-tail [199,200,201]. Like lncRNAs, circRNAs also cis- and transregulate target molecules with similar mechanisms, such as miRNA sponges and protein decoys, but have additional regulatory roles towards parental genes [199,200,201].

Although the information about circRNAs in NAFLD is scarce, there are some circRNAs that are known to regulate hepatic steatosis. Two circRNAs act as a sponge to miR-34a: *circRNA_0046366* [169] and *circRNA_0046367* [170]. Both circRNAs prevent the binding of miR-34a to PPARA and alleviate hepatic steatosis by restoring the lipid metabolism pathways and genes [169,170]. Another circRNA is *circRNA_021412* that acts as a sponge to miR-1972. This inhibition of miR-1972 causes an increase of Lipin 1 (*LPIN1*) expression, and LPIN1 is a co-activator of PPARA [173]. Therefore, the combined actions of *circRNA_021412*, *circRNA_0046366*, and *circRNA_0046367* to suppress the miRNAs that control PPARA signaling may be an alternative therapeutic target to reduce hepatic steatosis.

In NASH, an experimental study of a NASH animal model identified one circular RNA, *circRNA_002581*, as a central modulator in NASH [168]. This circRNA was higher in NASH mice, and the computational network analysis revealed that this circRNA could act as a miR-122 sponge. Inhibition of miR-122 action leads to the increased expression of three genes (*Slc1a5*, *Plp2*, and *Cpeb1*), validated with real-time PCR. All of these three genes are involved in NAFLD [168].

Some circRNAs regulate fibrosis. One such is *circUBE2K* from the parental gene of ubiquitin-conjugating enzyme E2 K (*UBE2K*). In a human HSC cell line study, *circUBE2K* was found to act as a sponge to miR-149-5p and increase *TGFB2* expression to initiate the expression of fibrosis genes (*ACTA2* and *COL1A1*) [176]. Another circRNA is *circRNA_0074410*, which acts as a sponge to miR-9-5p [172]. Although no target gene was identified in this study of the human HSC cell line [172], a previous study revealed that this miR-9-5p could regulate both *TGFB* receptors and suppress TGFB signaling [202]. *circPWWP2A* also regulates TGFB signaling, as this circRNA is a sponge to miR-203, thus removing the suppression of follistatin-like 1 (*FSTL1*) expression, and therefore, FSTL1 could interact with SMAD proteins to facilitate TGFB signaling [167]. Two circRNAs are sponges to miR-146a-5p, *circTUBD1* [175] and *circRSF1* [174]. A loss of miR-145a-5p causes the activation of HSCs via toll-like receptor 4 (*TLR4*) [175] and Ras-related C3 botulinum toxin substrate 1 (*RAC1*) [174]. Other circRNAs promote fibrosis via other pathways than the TGFB. One such is *circRNA_0067835*, which acts as a sponge to miR-155 and induces *FOXO3A* suppression [171]. Knockdown of *circRNA_0067835* caused a reduction of *FOXO3A* due to higher miR-155 expression and inhibition of HSC proliferation via the suppression of the PI3K/AKT pathway [171].

There are several circRNAs that negatively regulate fibrosis. An example is *circ_0007874*, or *cMTO1*, which acts as a sponge to miR-17-5p and increases *SMAD7* expression, a negative regulator of TGFB signaling [162]. Another circRNA that regulates *SMAD7* expression is *circPSD3*, which acts as a sponge to miR-92b-3p [166]. Besides the TGFB pathway, circRNAs could also alleviate hepatic fibrosis via a different pathway. Another study of *cMTO1* showed that *cMTO1* could act as a sponge to miR-181b-5p [163]. The suppression of miR-181b-5p action leads to higher expression of the phosphatase and tensin homolog (*PTEN*), a negative regulator of the PI3K/AKT pathway [163]. Similar to *cMTO1*, *circFBXW4* acts as a sponge to miR-181b-5p, and this suppression leads to higher expression of *SREBF1* and inhibition of the PI3K/AKT pathway [164,165]. Although information about the role of circRNAs in NAFLD is still new and limited, these findings showed that understanding these circRNA functions would be beneficial, as some of these circRNAs are pro-fibrotic and some are anti-fibrotic. Investigation of their diagnostic potential with respect to NAFLD in large and various population cohorts is also needed before this marker could be used in primary care settings.

### 4.3. Extracellular Vesicles in NAFLD

Inter-cellular communication is not limited to direct contact in cell-to-cell interaction, there being adjacent communication via the secretion of information-bearing membrane lipid vesicles known as extracellular vesicles (EVs) [203]. Generally, EVs are grouped according to their sizes and biogenesis as (1) exosomes (the smallest, 30–150 nm, generated from the intraluminal vesicles within multi-vesicular bodies (MVBs) fused with the plasma membrane), (2) microvesicles (MV, 50–1000 nm, formed by outward budding of the plasma membrane), and (3) apoptotic bodies (100–5000 nm, produced when membrane blebbing occurs during apoptosis) [203].

Although the research on EVs in NAFLD is still new, the findings show that EVs could play significant roles in NAFLD progression, as summarized previously [203,204]. For example, lipotoxicity in hepatocytes could lead to cell apoptosis and trigger inflammation. Thus, lipotoxic hepatic EVs could be the main drivers for the NAFLD progression to NASH. Lipotoxic hepatocytes could initiate macrophage activation to an inflammatory phenotype (M1) through the NFKB pathway [205]. Excessive lipid accumulation initiated death receptor 5 (DR5)/caspase signaling which activates Rho-associated protein kinase 1 (*ROCK1*) and subsequently enriches the TRAIL receptor on EVs to initiate macrophage activation [205]. Another example is nucleotide-binding oligomerization domain-like receptor protein 3 (NLRP3) inflammasome activation. Lipotoxic hepatic EVs could activate the NLRP3 inflammasome in macrophages and neighboring hepatocytes [206]. Mechanistically, the lipotoxic hepatic EVs may contain molecules or modulators to initiate inflammatory responses in other cells. This mechanism is partly understood as hepatic EVs were enriched with miR-192-5p which activated macrophage M1 polarization via RICTOR/AKT/FOXO1 signaling inhibition [207]. EVs also could modulate fibrosis formation. One example is that lipotoxic hepatic EVs could be internalized by the HSCs and cause the activation of HSCs. In a study of a mouse model of steatosis, these lipotoxic hepatic EVs have enrichment of miR-128-3p in their vesicles, and this miRNA regulates multiple fibrosis-related genes, including *Pparg* [208]. Another study of hepatic excessive lipid accumulation revealed that lipotoxic hepatic EVs have higher levels of miR-122 and miR-192 in their vesicles. These miRNAs initiate HSC activation by increasing the expression of fibrosis-related genes [209].

Although EVs are detectable in circulating biofluids, their origins are essential to determine the specificity of the disease. A recent study of a mouse model of NAFLD showed that hepatic EVs could be isolated using nanoscale flow cytometry by detecting the surface markers of asialoglycoprotein receptor 1 (ASGR1) and cytochrome P450 family 2 subfamily E member 1 (CYP2E1) on the EVs [210]. In this study of the NASH mouse model [210], there was an enrichment of hepatic EVs in mice with early signs of NAFLD before the histological appearance of liver inflammation. The levels of these EVs remained high until the end of the study and correlated strongly with the NASH parameters [210]. Since this area of research is still new, the information about the diagnostic potential of these EVs for NASH and NAFLD identification is still limited, though the prospect is promising.

### 4.4. Metabolomics in NAFLD

Another potential area for developing a diagnostic tool for NAFLD and NASH is the metabolomics of NAFLD. Metabolomics is a study of all metabolites, the small molecules, intermediates, and products of cell metabolism [211,212]. A recent systematic review of 11 studies showed that half of these studies reported higher levels of circulating branched-chain amino acids (BCAAs), including leucine, isoleucine, and valine in NAFLD [211]. Enrichment of these BCAAs could activate the mTOR pathway, causing the inhibition of fatty acid conversion to triglycerides and increasing lipid accumulation (steatosis) [213]. Besides BCAAs, the levels of phosphatidylcholine (PC) and sphingolipids are lower in NAFLD [211]. A low level of PC is related to adipocyte turnover, in which the size of the adipocytes was higher to compensate for the need for higher lipid storage [214], thus indicating that the metabolites could also reflect the status of adipocytes. Recently, metabolomic profiling of NAFLD patients at different stages showed that several metabolites increase with the severity of NAFLD. Glycocholic acid, taurocholic acid, phenylalanine, and BCAAs all increase according to severity from steatosis to NASH and NASH to cirrhosis. Notably, an ensemble machine learning (EML) model built to handle these metabolites could diagnose NAFLD with more than 80% accuracy [215]. Although the findings are limited, the potential of metabolomics for diagnosing NAFLD and its stages is promising enough for future validation.

## 5. Conclusions and Future Directions

Almost all current tools and technologies for diagnosing NAFLD could diagnose advanced fibrosis and cirrhosis well, though gaps remain with respect to the identification of good markers for NASH and early fibrosis. Liver biopsy remains the gold-standard method to assess NASH; however, the development of noninvasive tools to limit or avoid the usage of liver biopsy has become a research priority. Therefore, understanding and identification of biomarkers specific to different stages of NAFLD, notably NASH, are of great importance. Alternative biomarkers, such as circulating noncoding RNAs, genetic markers, and extracellular vesicles, show significant potential. Therefore, these most promising new biomarkers should be further developed and validated in various populations.

## Figures and Tables

**Table 1:** Summary of the serum or plasma biomarker indexes used to diagnose NAFLD.

Index Models	Clinical Markers	Serum or Blood Markers	Reference
Steatosis			
FLI	BMI, WC	GGT, TG	[31]
HSI	BMI, Diabetes Status	AST/ALT ratio	[32]
SteatoTest	Age, Sex, BMI	ALT, GGT, TG	[33]
LAP	Age, Sex, BMI, WC	ALT, AST, GGT, Glucose level, TG	[34]
ION	Sex, Waist-to-hip ratio, Diabetes status	TG, ALT, HOMA-IR	[35]
NAFLD-LFS	Diabetes and MetS status	Serum-insulin, AST/ALT ratio	[36]
TyG	Age, Sex, BMI, SBP, DBP	HbA1c, Uric acid, HDL-C	[37]
VAI	Age, BMI, PCOS diagnosis	ALT, GGT, TG, DHEA-S, SHBG, HOMA-IR	[39]
NASH			
HAIR	Waist-to-hip ratio	ALT, TG, FP-insulin, FP-glucose, C-peptide levels	[41]
Palekar score	Age, Sex, BMI,	AST, AST/ALT ratio, Fasting-insulin, QUICKI, HA	[42]
oxNASH	Age, BMI	9- & 13-HODEs, 9- & 13-oxoODEs, Free-radical mediated oxidation of LA	[43]
Gholam score	Diabetes and MetS status	ALT, AST, GGT, HbA1c, TG,	[44]
NAFIC score	Age, Sex, Diabetes status	Serum ferritin, Fasting-insulin, Immunoreactive insulin, Type IV collagen 7S	[45]
NashTest	Age, Sex, Height, Weight	Alpha2macroglobulin, Apolipoprotein A1, AST, Cholesterol, Haptoglobin, GGT, TG, Total bilirubin Transaminases ALT	[46]
NASH Score	Age, Sex, BMI, Diabetes status	AST, Fasting-insulin and circulating CK-18 fragment concentrations, *PNPLA3* genotype	[47]
NASH ClinLipMet Score	Age, Sex, BMI, MetS status	AST, Fasting-insulin, Glu, Gly, Ile, LysoPC16:0, PE40:6, TG48:0, Ser, *PNPLA3* genotype	[48]
acNASH	Age	AST, SCr	[49]
Fibrosis			
NFS	Age, BMI, Hyperglycemia, Diabetes, Hypertension status	Albumin, Platelet count, AST/ALT ratio	[50]
BARD	BMI, Diabetes status	AST/ALT ratio	[51]
APRI	Age, Diabetes status	ALP, AST, Platelet count	[52]
FIB-4	Age	ALT, AST, INR, Platelet count	[53]
FibroTest	Age, Sex	Alpha-2 macroglobulin, Apolipoprotein A_1_, GGT, Gamma-globulin, Haptoglobin, Total bilirubin	[54]
FibroMeter	Body weight, MetS status	ALT, AST, Ferritin, Glucose, Platelet count	[55]
ELF	Age, Sex	Collagen IV (T59106R), Collagen VI, HA, laminin, MM2, MM9, PIIINP, TIMP-1, Tenascin	[56]
Hepascore	Age, BMI, Diabetes status	Aminoterminal peptide of procollagen-III, HA, TIMP-1	[57]

Abbreviation: ALT: alanine transaminase; ALP: alkaline phosphatase; APRI: aspartate transaminase-to-platelet ratio index; AST: aspartate transaminase; BMI: body mass index; DHEA-S: dehydroepiandrosterone sulphate; DBP: diastolic blood pressure; FP: fasting plasma; FLI: fatty liver index; GGT: gamma-glutamyltransferase; HbA1c: hemoglobin A1c; Glu: glutamate; Gly: glycine; glycosylated hemoglobin A1c; HA: hyaluronic acid; HDL-C: high-density lipoprotein cholesterol; HIS: hepatic steatosis index; HODE: hydroxy-octadecadenoic acids; HOMA-IR: homeostasis model assessment of insulin resistance; Ile: isoleucine; IRI: immunoreactive insulin; LA: linoleic acid; LAP: lipid accumulation product; LysoPC16:0: lysophosphatidylcholine; MetS: metabolic syndrome; MM: matrix metalloproteinase; NFS: NAFLD fibrosis score; NAFLD-LFS: NAFLD liver fat score; NASH: nonalcoholic steatohepatitis; oxoODE: oxo-octadecadenoic acids; PCOS: polycystic ovary syndrome; PE40:6: phosphoethanolamine 40:6; PIIINP: N-terminal propeptide of type III collagen; QUICKI: quantitative insulin sensitivity check index; SBP: systolic blood pressures; SCr: serum creatinine; Ser: serine; SHBG: sex hormone binding globulin; TG: triglycerides; TIMP-1: tissue inhibitor of matrix metalloproteinase 1; TyG: triglyceride-glucose index; VAI: visceral adiposity index; WC: waist circumstances.

**Table 2:** Summary of the noncoding RNAs in NAFLD.

Noncoding RNAs	Target Molecules	Expression	Role in NAFLD	Reference
MicroRNAs				
miR-122	*SREBF1*, *DGAT2*, *FASN, P4HA1*	High	Steatosis, liver fibrosis	[127]
miR-138, -143	*BCL2, TGFB*	High	Liver fibrosis	[128]
miR-181b	*PTEN*	High	Liver fibrosis	[129]
miR-192	*ALCAM*, *EREG*, *MSN*, *Zeb2*	Low	Liver fibrosis	[130,131]
miR-21	*Foxa2, Foxo1, Hnf4a, Stat3, Ppara*	High	Steatosis	[132,133,134,135]
miR-221	*COL1A1*	High	Liver fibrosis	[136]
miR-29a	*TGFB, NFKB*	Low	Liver fibrosis	[137]
miR-33a,b	*SREBF1, SREBF2*	High	Steatosis	[138]
miR-34a-5p	*Ppara, Sirt1*	High	Steatosis	[139,140]
miR-99a	*TNF, mTOR/SREBF1*	Low	Steatosis	[141,142]
LncRNAs				
*APTR*	*PRC2*	High	Liver fibrosis	[143]
*FLRL2*	*ARNTL*	Low	Inflammation, steatosis	[144]
*GAS5*	miR-222	High	Liver fibrosis	[145]
*H19*	*Ptbp1l*, *Srebf1*	High	Steatosis	[146]
*HIF1A-AS1*	*TET3*	Low	Liver Fibrosis	[147]
*HOTAIR*	miR-29b, *DNMT1, PRC2*	High	Liver fibrosis	[148,149]
*HOTTIP*	miR-148a	High	Cirrhosis	[150]
*HULC*	*MAPK*	High	Liver fibrosis	[151]
*LFAR1*	Smad2/3, Tgfbr1	High	Liver fibrosis	[152]
*LncRNA-ATB*	miR-200a, *CTNNB1*	High	Liver fibrosis	[153]
*LncRNA-P21*	miR-181b, miR-17-5p	High	Liver fibrosis	[129,154]
*LncSHGL*	*Hnrnpa1*	Low	Steatosis	[155]
*MALAT1*	*Srebf1*	High	Inflammation, liver fibrosis	[156]
*MEG3*	*TP53*, miR-21	High	Liver fibrosis	[157,158]
*SRA*	*Foxo1, Pparg*	High	Steatosis	[159]
*SCARNA10*	*PRC2*	High	Liver fibrosis	[160]
*TUG1*	miR-29b	High	Cirrhosis	[161]
CircRNAs				
*cMTO1*	miR-17-5p/*SMAD7,* miR-181b-5p/*PTEN*	Low	Liver fibrosis	[162,163]
*circFBXW4*	miR-181b-5p, *SREBF1*	Low	Liver fibrosis	[164,165]
*circPSD3*	miR-92b-3p, *SMAD7*	Low	Liver fibrosis	[166]
*circPWWP2A*	miR-203, *FSTL1*	High	Liver fibrosis	[167]
*circRNA_002581*	miR-122, *Slc1a5*, *Plp2*, *Cpeb1*	High	NASH	[168]
*circRNA_0046366*	miR-34a, *PPARA*	Low	Steatosis	[169]
*circRNA_0046367*	miR-34a, *PPARA*	Low	Steatosis	[170]
*circRNA_0067835*	miR-155, *FOXO3A*	High	Liver fibrosis	[171]
*circRNA_0074410*	miR-9-5p	Low	Liver fibrosis	[172]
*circRNA_021412*	miR-1972, *LPIN1*	Low	Steatosis	[173]
*circRSF1*	miR-146a-5p, *RAC1*	High	Liver fibrosis	[174]
*circTUBD1*	miR-146a-5p, *TLR4*	High	Liver fibrosis	[175]
*circUBE2K*	miR-149-5p, *TGFB2*	High	Liver fibrosis	[176]

Abbreviation: ALCAM: activated leukocyte cell adhesion molecule; APTR: Alu-mediated p21 transcriptional regulator; ARNTL: aryl hydrocarbon receptor nuclear translocator-like protein 1; BCL2: B-cell lymphoma 2; CircRNA: circular RNA; COL1A1: alpha-1 type I collagen; CPEB1: cytoplasmic polyadenylation element binding protein 1; CTNNB1: beta catenin; DGAT2: diacylglycerol O-acyltransferase; DNMT1: DNAmethyl transferase 1; EREG: epiregulin; FASN: fatty acid synthase; FLRL2: fatty liver-related lncRNA 2; FOXA2: forkhead box transcription factor A2; FOXO1: forkhead box transcription factor O1; FSTL1: Follistatin-like 1; GAS5: growth arrest-specific 5; H19: H19 imprinted maternally expressed transcript; HIF1A-AS1: HIF1A antisense RNA 1; HNF4A: hepatocyte nuclear factor 4 alpha; HOTAIR: HOX transcript antisense RNA; HOTTIP: HOXA transcript at the distal tip; HULC: highly upregulated in liver cancer; LFAR1: liver fibrosis-associated lncRNA 1; LncRNA: long noncoding RNA; LncRNA-ATB: long noncoding RNA activated by TGFB; LPIN1: Lipin 1; MALAT1: metastasis-associated lung adenocarcinoma transcript 1; MAPK: mitogen-activated protein kinase; MEG3: maternally expressed 3; MIRT2: myocardial infarction-associated transcript 2; MSN: moesin; mTOR: mammalian target of rapamycin; NFKB: nuclear factor kappa-light-chain-enhancer of activated B cells; P4HA1: prolyl 4-hydroxylase subunit alpha 1; PLP2: proteolipid protein 2; PPARA: peroxisome proliferator-activated receptor alpha; PPARG: peroxisome proliferator-activated receptor gamma; PRC2: polycomb repressive complex 2; PTBP1L: Polypyrimidine tract-binding protein 1; PTEN: phosphatase and tensin homolog; RAC1: Ras-related C3 botulinum toxin substrate 1; SCARNA10: small cajal body-specific RNA 10; SIRT1: sirtuin 1; SMAD: SMAD family members; SLC1A5: solute carrier family 1 member 5; SRA: steroid receptor RNA activator; SREBF1: sterol regulatory element-binding transcription factor 1; STAT3: signal transducer and activator of transcription 3; TET3: ten-eleven-translocation 3; TGFB: transforming growth factor beta; TNF: tumor necrosis factor; TGFBR1: TGFB receptor; TLR4: toll-like receptor 4; TP53: tumor suppressor p53; TUG1: taurine up-regulated 1; UBE2K: ubiquitin-conjugating enzyme E2 K; ZEB2: zinc finger E-box binding homeobox 2.

## Data Availability

Not applicable.
